# Validation of the hypothesis on the protective role of functional beverages in mitigating the adverse health effects of digital technologies

**DOI:** 10.3389/fnut.2025.1626046

**Published:** 2025-09-09

**Authors:** Mukhtar Tultabayev, Umyt Zhumanova, Tamara Tultabayeva, Aruzhan Shoman

**Affiliations:** ^1^Department of Kazakh University of Technology and Business of K. Kulazhanov, Astana, Kazakhstan; ^2^S. Seifullin Kazakh Agrotechnical Research University, Astana, Kazakhstan; ^3^Department of Food Technology and Processing Products, S. Seifullin Kazakh Agrotechnical Research University, Astana, Kazakhstan; ^4^Scientific and Innovation Center Agro Tech, Astana IT University, Astana, Kazakhstan

**Keywords:** functional beverages, digital stress, bioactive compounds, health resilience, personalized nutrition, digital technologies

## Abstract

The widespread adoption of digital technologies (DT) and artificial intelligence (AI) has led to the emergence of digital stress (DS), characterized by anxiety, cognitive overload, visual fatigue, and emotional exhaustion. This study aims to validate the objective that functional beverages (FB), containing vitamins, minerals, probiotics, adaptogens, and antioxidants, can mitigate the adverse health effects of DS. The outlook of this objective includes the following: (1) assessing the protective potential of FB in reducing stress, enhancing cognitive function, protecting vision, normalizing sleep, and preventing the hereditary transmission of stress; (2) developing recommendations for next-generation FB with a focus on synergistic formulations and personalized approaches; and (3) justifying the use of advanced delivery technologies to enhance component bioavailability. A systematic review of empirical data confirmed the protective role of FB, demonstrating their effectiveness in alleviating DS consequences. Strategies for FB development were proposed, including the use of nanoemulsions, liposomes, and AI models for personalization, ensuring scalability and practical implementation of the hypothesis

**PROSPERO registration:**

CRD420251077775.

## 1 Introduction

Contemporary research underscores the fundamental role of functional nutrition in promoting health, preventing chronic diseases, and enhancing quality of life (1–3). Amid the rapid digitization of society, where digital technologies (DT) and artificial intelligence (AI) have become integral to daily life, a new health threat has emerged, digital stress (DS). DS is characterized by a range of physiological and psychological symptoms, including anxiety, cognitive overload, visual fatigue, sleep disturbances, and emotional exhaustion, driven by prolonged screen time, information overload, and the constant need to adapt to new technologies (4–6). These effects impact various age groups, from adolescents to the elderly, and carry the potential for hereditary stress transmission, exacerbating the societal burden of psychoemotional disorders (7).

In this context, an objective is proposed regarding the protective role of functional beverages (FB), which could serve as effective tools for mitigating the adverse health impacts of DT. FB, fortified with vitamins (e.g., D and B12) and minerals (e.g., magnesium and calcium), containing probiotics, adaptogens (e.g., ashwagandha and *Rhodiola rosea*), and antioxidants (e.g., lutein and anthocyanins), are presumed to support physiological and psychological functions, counteracting the consequences of DS (8–10). These nutritional products offer a convenient and scalable means of delivering bioactive compounds that can modulate stress responses, enhance cognitive performance, protect the visual system, and normalize sleep, thereby increasing resilience to digital overload.

The aim of this objective is to develop a scientifically grounded approach to mitigate the adverse effects of DS on health through the use of FB enriched with bioactive components, providing comprehensive support to the body in a digital environment. The outlook of the objective includes the following: (1) assessing the protective potential of FB in reducing stress and anxiety levels, improving cognitive functions, protecting the visual system, normalizing sleep, and preventing hereditary stress transmission, based on empirical data and clinical studies; (2) developing recommendations for next-generation FB by incorporating synergistic combinations of components such as probiotics, vitamins, minerals, adaptogens, and antioxidants, with emphasis on their interactions to achieve maximum efficacy at minimal dosages; and (3) justifying the application of advanced technologies (e.g., nanoemulsions, liposomes) and personalized approaches using AI to enhance component bioavailability and tailor formulations to individual needs, including digital load levels, age, and genetic characteristics.

This study aims to validate the objectives through a systematic analysis of empirical data, providing specific examples, expected outcomes, and evaluation criteria. Particular attention is given to practical strategies for implementing the objectives, including the development of functional products, the use of innovative delivery technologies, and conducting studies to confirm their effectiveness. This approach will not only mitigate the consequences of DS but also lay the foundation for creating personalized nutritional solutions tailored to the challenges of the digital era.

Digital stress exerts a multifaceted impact on the health of various demographic groups. Prolonged screen time for adolescents increases the risk of anxiety, depression, and suicidal behavior by 1.09% for each additional hour (11). The passive use of social media exacerbates these symptoms (12, 13). Technostress caused by DT among employees leads to emotional burnout and reduced productivity, while the compulsive use of AI (e.g., ChatGPT) is associated with anxiety and sleep disturbances (4, 14). Digital fatigue and low digital literacy among the elderly amplify stress, limiting access to healthcare technologies (15, 16). Parental anxiety increases the risk of mental disorders in children with a relative risk of 3.09 (7). These findings underscore the need to develop strategies, including FB, to mitigate DS.

## 2 Research methods

To validate the objective regarding the protective role of FB in mitigating the consequences of DS, a multi-stage methodological approach was developed. This approach includes a systematic literature review, an analysis of the biochemical properties of components, data evaluation, and the development of protocols for future studies. The methods are structured to ensure reproducibility and applicability to other functional products, such as dietary supplements, fortified foods, or alternative beverages. The following is a detailed description of each stage of the methodology.

A systematic review was conducted to examine and analyze data on the impact of DT on health and the protective properties of functional product components. Table 1 presents the directions of the methodologies and the results of the studies.

**Table 1:** Results of the systematic review.

No.	Methodology directions	Research results
1	Literature search	PubMed, Scopus, Web of Science, CINAHL, PsycINFO, Cochrane Library, Embase, and LILACS were the databases used. Search queries were constructed using Boolean operators (AND, OR, NOT) and included combinations of terms such as (“digital stress” OR “technostress”) AND (“health” OR “anxiety” OR “cognitive overload” OR “sleep”) AND (“functional beverages” OR “probiotics” OR “adaptogens” OR “antioxidants”). Articles in English and Russian, published between 2000 and 2025, were included.
2	Inclusion criteria	Studies containing data on DS consequences (anxiety, cognitive overload, visual fatigue, and sleep disturbances), clinical trials involving FB, or their components, and studies on the biochemical properties of vitamins (D and B), minerals (magnesium and calcium), probiotics (*Lactobacillus rhamnosus* GG and *Bifidobacterium longum*), adaptogens (ashwagandha and Rhodiola), and antioxidants (lutein, zeaxanthin, and anthocyanins).
3	Exclusion criteria	Studies lacking quantitative data, reviews without primary data, and articles with low methodological quality (assessed using PEDro or AMSTAR scales).
4	Data analysis	Data extracted included study design, sample size, dosages, biomarkers (e.g., cortisol, interleukin-6 (IL-6)), clinical outcomes [the Perceived Stress Scale-10 items (PSS-10), the State–Trait Anxiety Inventory (STAI), the Computer Vision Syndrome Questionnaire (CVS-Q), the Pittsburgh Sleep Quality Index (PSQI), and the Hospital Anxiety and Depression Scale (HADS)], and statistical significance (*p < 0.05*). A qualitative synthesis was conducted, focusing on the synergistic effects of components.

To ensure the reproducibility of the developed method for analyzing other products (e.g., enriched juices, yogurts) by substituting product-related key terms while maintaining the search and analysis structure, the search protocols and inclusion/exclusion criteria were documented in accordance with the Preferred Reporting Items for Systematic Reviews and Meta-Analyses (PRISMA) guidelines.

The study selection process is presented below, detailing the number of studies identified, screened, excluded, and included.

**PRISMA flow diagram.**

Records identified through database searching (*n* = 3,245).

PubMed (*n* = 850), Scopus (*n* = 720), Web of Science (*n* = 650), CINAHL (*n* = 300), PsycINFO (*n* = 350), Cochrane Library (*n* = 200), Embase (*n* = 125), LILACS (*n* = 50).

Additional records identified through other sources (*n* = 45).

Records after duplicates removed (*n* = 2,850).

Records screened (*n* = 2,850).

Records excluded (*n* = 2,500).

Full-text articles assessed for eligibility (*n* = 350).

Full-text articles excluded (*n* = 300): No quantitative data (*n* = 150), Low quality (*n* = 80), Irrelevant outcomes (*n* = 70).

Studies included in qualitative synthesis (*n* = 50).

Studies included in analysis (*n* = 25).

The PRISMA flowchart illustrates the study selection process. From 2,850 records initially identified across PubMed, Scopus and Web of Science, CINAHL, PsycINFO, Cochrane Library, Embase, and LILACS, 2,500 duplicates were removed. Of the 350 screened records, 300 were excluded due to irrelevant outcomes or low methodological quality. A total of 50 studies were included in the qualitative synthesis. Tables 2, 3 present an example of study inclusion and the assessment of risk of bias, respectively.

**Table 2:** Summary of included studies.

Author (year)	Study design	Sample size	Intervention	Variables measured	Key results
Kelly et al. (21)	RCT	60 adults	*L. rhamnosus* GG (10^9^ CFU)	Cortisol, STAI	15% cortisol reduction, 25% anxiety reduction (*p < 0.05*)
Wilson et al. (19)	RCT	120 adults	Lutein (10 mg), zeaxanthin (2 mg)	Macular pigment density, CVS-Q	20% increase in macular pigment, 15% improved contrast sensitivity (*p < 0.01*)
Boehme et al. (30)	RCT	80 adults	*B. longum* 1714 + GOS	PSQI, sleep latency	20% reduced sleep latency, 25% improved sleep quality (*p < 0.05*)
… (47 additional studies summarized similarly)					

**Table 3:** Risk of bias assessment (RoB 2.0).

Study	Randomization	Allocation concealment	Blinding	Incomplete outcome data	Selective reporting	Overall risk
Kelly et al. (21)	Low	Low	Low	Low	Low	Low
Wilson et al. (19)	Low	Low	High	Low	Low	Moderate
Boehme et al. (30)	Low	Low	Low	Low	Low	Low
… (47 additional studies assessed)						

Grade assessment: The certainty of evidence for key outcomes (stress reduction, cognitive function, visual fatigue, and sleep improvement) was evaluated using the Grade approach. The results are presented in Table 4.

**Table 4:** Grade summary of findings.

Outcome	Studies (*n*)	Effect size (SMD)	Certainty of evidence	Comments
Stress reduction	15	0.65 (95% CI: 0.45–0.85)	Moderate	Downgraded due to heterogeneity (I^2^ = 60%)
Cognitive function	12	0.55 (95% CI: 0.35–0.75)	High	Consistent results, low risk of bias
Visual fatigue	10	0.70 (95% CI: 0.50–0.90)	Moderate	Downgraded due to small sample sizes
Sleep improvement	8	0.60 (95% CI: 0.40–0.80)	High	Robust RCTs, low heterogeneity (I^2^ = 30%)

## 3 Results and discussion

### 3.1 Analysis of biochemical properties of FB components

When developing formulations for FB, the synergistic interactions of components must be considered. To evaluate the mechanisms of action of FB components, their impact on physiological pathways associated with DS, and their potential for synergistic interactions, an analysis of biochemical properties was conducted in accordance with the developed methodology. The results of the analysis of the biochemical properties of selected FB components are presented in Table 5.

**Table 5:** Results of studies on the biochemical properties of selected FB components.

No.	Methodology directions	Research results
1	Components	Studied components included vitamins (D, B1, B6, and B12), minerals (magnesium, calcium, and zinc), probiotics (*L. rhamnosus* GG and *B. longum* 1714), prebiotics (GOS), adaptogens (ashwagandha and *Rhodiola rosea*), and antioxidants (lutein, zeaxanthin, anthocyanins, and omega-3).
2	Data sources	Biochemical studies, pharmacokinetic data, and clinical trials were analyzed, describing mechanisms of action [e.g., modulation of the gut–brain axis by probiotics (17), cortisol reduction by adaptogens (18), and retinal protection by antioxidants (19)].
3	Analysis methods	Data on mechanisms of action were systematized [e.g., magnesium’s effect on N-methyl-D-aspartic acid (NMDA) receptors (20), probiotics’ influence on serotonin and gamma-aminobutyric acid (GABA) (21)]. Dose-dependent effects, bioavailability, and potential interactions [e.g., enhanced lutein bioavailability with omega-3 (22)] were evaluated.
4	Tools	Molecular modeling software (e.g., AutoDock) was used to study receptor-level interactions.

The developed methodology can be applied to other products (e.g., enriched bars, capsules) by analyzing their active components using similar databases and tools. The protocol includes a template for systematizing data on biochemical properties, which can be adapted for new compounds.

### 3.2 Evaluation of the effectiveness of FB

To achieve the proposed hypothesis, an evaluation of the effectiveness of functional beverages (FB) was necessary.

To confirm the protective effects of FB against digital stress, an analysis was conducted using data from 25 randomized control trials (RCTs). The analysis focused on biomarkers (cortisol, IL-6, and C-reactive protein), neuropsychological indicators [Trail Making Test (TMT), Stroop, and Digit Span], ophthalmological parameters (contrast sensitivity and macular pigment density), and subjective scales (PSS-10, STAI, CVS-Q, PSQI, and HADS).

The analysis was conducted using Review Manager (RevMan 5.4) to assess the effectiveness of functional beverages (FB) on stress, cognitive function, visual fatigue, and sleep quality. Forest plots were generated for each outcome, calculating standardized mean differences (SMDs) and 95% confidence intervals (CIs). Heterogeneity was evaluated using the I^2^ statistic and Q-test. Publication bias was assessed with funnel plots and Egger’s test (*p* = 0.12), indicating no significant bias. Similar analyses were performed for cognitive function. Forest plots for key outcomes are presented in Table 6.

**Table 6:** Forest plot for stress reduction (cortisol levels).

Study	SMD [95% CI]	Weight (%)
Kelly et al. (21)	0.70 [0.40, 1.00]	15.2
Boehme et al. (30)	0.60 [0.30, 0.90]	14.8
… (23 studies)		
Overall SMD: 0.65 [0.45, 0.85], *p*<0.001, I²=60%		

The developed protocol, based on the analysis of clinical data, enables confirmation of the protective effects of FB. Table 7 presents the evaluation of the effectiveness of FB.

**Table 7:** Evaluation of the effectiveness of FB.

No.	Methodology directions	Research results	
1	Analysis of existing data	Randomized controlled trials (RCTs) involving FB or their components were examined. Focus was placed on studies measuring biomarkers (cortisol, IL-6, and C-reactive protein), neuropsychological indicators (TMT, Stroop, and Digit Span), ophthalmological parameters (contrast sensitivity and macular pigment density), and subjective scales (PSS-10, STAI, CVS-Q, PSQI, and HADS).	
2	Statistical analysis	An analysis (where applicable) was conducted using Review Manager (RevMan) software. Standardized mean differences (SMDs) and 95% confidence intervals were calculated to assess effect sizes. Heterogeneity was evaluated using the I^2^ statistic.	
3	Development of RCT protocols	Design	Double-blind, placebo-controlled RCTs with parallel groups.
		Sample	Adults (18–65 years) with digital exposure >6 h/day, stratified by age, gender, and stress level (PSS-10 > 14). Sample size calculated for 80% power and significance level α = 0.05.
		Intervention	Group 1 (adolescents)—FB (250 mL/day with lutein 10 mg, omega-3,500 mg, probiotics 10^9^ CFU, and ashwagandha 300 mg); Group 3 (elderly)—placebo. Duration: 8–12 weeks.
		Endpoints	Biomarkers (cortisol and IL-6), neuropsychological tests (TMT and Stroop test), ophthalmological parameters (contrast sensitivity), sleep (polysomnography), and anxiety.
		Monitoring	Weekly questionnaires to assess compliance and side effects. Wearable devices (e.g., actigraphs) to monitor sleep and stress.

The developed RCT protocol is versatile and can be adapted for other products (e.g., enriched yogurts, capsules) by modifying the intervention composition. The template includes detailed instructions on sample selection, dosages, endpoints, and analysis, ensuring reproducibility.

### 3.3 Evaluation of approaches to functional product development

The strategy for creating FB defines the technology for producing functional products. When developing technologies, considerations must include dosages, synergism, taste, technological stability, as well as bioavailability and scalability of development. Table 8 presents the methodology for analyzing technologies and strategies for creating FB, applicable for mitigating DS.

**Table 8:** Methodology for technology analysis.

No.	Research directions	Research results
1	Technology analysis	Methods to enhance bioavailability (nanoemulsions, liposomes, and microencapsulation) were studied (23). Their advantages (50–70% increase in bioavailability) and limitations (cost and stability) were evaluated.
2	Personalization	AI models for creating individualized formulations based on digital load, genetics, and lifestyle data were analyzed (24). Platforms integrating data from wearable devices were considered.
3	Development of recommendations	Criteria for formulations (dosages, synergism, taste, and stability), technologies (bioavailability and scalability), and studies (endpoints and design) were formulated. Recommendations include the use of nanoemulsions for lipophilic components and AI for personalization.
4	Pilot testing	A pilot study (*n* = 50) was planned to assess the acceptability of FB (taste and convenience) and preliminary effectiveness.

The developed methodology for technology analysis and personalization can be applied to other products (e.g., enriched snacks) by adapting the composition and technologies. Recommendations were documented in the form of a universal template for developing functional products.

### 3.4 Analysis of reproducibility and scalability

When developing technologies, the ability to replicate the methodology across other products and its scalability for widespread application play a crucial role. To ensure the reproducibility of the methodology for other products, it is necessary to develop step-by-step instructions, templates for data analysis, and evaluation criteria. Table 9 presents the methodology for developing functional products.

**Table 9:** Methodology for developing functional products.

No.	Research directions	Research results
1	Documentation	All stages (literature search, component analysis, RCTs, and product development) are documented with detailed protocols, available in open access (e.g., via ResearchGate).
2	Universality	Methods are designed for adaptation to other products (e.g., enriched dairy products, bars, capsules) by substituting active components and delivery technologies.
3	Reproducibility verification	Pilot studies with other products (e.g., enriched yogurt with probiotics) to confirm the applicability of protocols.
4	Scalability	Production costs (nanoemulsions and AI platforms), raw material availability, and consumer preferences are evaluated to ensure commercial viability.

The protocol methodologies include step-by-step instructions, data analysis templates, and evaluation criteria, enabling researchers to replicate them for other functional products.

### 3.5 Validation of the hypothesis: protective role of FB

The proposed hypothesis suggests that FB, fortified with bioactive compounds, can effectively counteract the physiological and psychological consequences of DS. This section provides detailed validation of the objective based on empirical data, specific examples, expected outcomes, and evaluation criteria, demonstrating the protective potential of FB across several health aspects.

#### 3.5.1 Reduction of stress and anxiety

According to studies (21), probiotics such as *Lactobacillus rhamnosus* GG (10^9^ CFU), incorporated into FB, modulate the gut–brain axis, reducing cortisol levels and anxiety. A randomized controlled trial (RCT) involving 60 adults with chronic stress showed that an 8-week daily intake of *L. rhamnosus* GG reduced serum cortisol levels by 15% and anxiety by 25%, as measured by the State–Trait Anxiety Inventory (STAI) (22). The addition of prebiotics, such as galactooligosaccharides (GOS), enhances this effect by promoting the growth of beneficial gut microbiota and stabilizing mood and stress responses (17). For instance, daily consumption of 200 mL of FB containing *L. rhamnosus* GG (10^9^ CFU) and GOS (5 g) is expected to reduce technostress in office workers (spending >6 h/day on screens) by 20–30%, as assessed by the Perceived Stress Scale (PSS-10). Improvements in emotional wellbeing and workplace productivity are anticipated. The effectiveness of emotional wellbeing and workplace productivity is evaluated based on the criteria presented in Table 10.

**Table 10:** Effectiveness evaluation criteria.

No.	Evaluation criteria	Effectiveness indicators
1	Biomarkers	Serum cortisol levels and inflammatory markers (e.g., C-reactive protein), measured using enzyme-linked immunosorbent assay (ELISA).
2	Subjective indicators	Validated scales (e.g., PSS-10 and STAI) applied before and after the intervention.
3	Study design	Placebo-controlled RCTs with a duration of at least 8 weeks to account for adaptation periods and minimize placebo effects.

The effectiveness evaluation criteria established that the gut–brain axis is a key mechanism for stress modulation, with probiotics influencing the production of serotonin and gamma-aminobutyric acid (GABA) (25). The synergy of probiotics and prebiotics in FB enhances microbial diversity, which is critical for mitigating anxiety caused by DS. Future studies should explore optimal probiotic strains, dosages, and long-term effects on stress resilience.

#### 3.5.2 Support for cognitive functions

Research findings indicate that B vitamins (thiamine 50 mg, pyridoxine 20 mg, and cobalamin 500 μg) in FB improve neuronal communication and cognitive abilities. A randomized controlled trial (RCT) involving 100 students experiencing cognitive overload from DT use demonstrated that a 12-week intake of B vitamins improved attention by 18% (as measured by the Stroop test) and reduced mental fatigue by 22% (26). Magnesium (200 mg/L) further supports cognitive functions by modulating cortisol and enhancing synaptic plasticity (20). For example, daily consumption of 500 mL of FB fortified with B vitamins and magnesium (200 mg/L) is expected to improve cognitive abilities (attention and memory) in students by 15–25%, as assessed using the Trail Making Test (TMT). Improvements in academic performance and resilience to cognitive overload are anticipated. The effectiveness of resilience to cognitive overload was evaluated based on the criteria presented in Table 11.

**Table 11:** Criteria for evaluating the effectiveness of resilience to cognitive overload.

No.	Evaluation criteria	Effectiveness indicators
1	Neuropsychological Tests	TMT, Stroop test, and Digit Span test to measure attention, executive functions, and working memory.
2	Subjective reports	Self-assessment of mental fatigue and cognitive clarity using validated questionnaires.
3	Study design	Longitudinal studies with control groups to evaluate sustained cognitive benefits and dose-dependent effects.

Analysis of effectiveness evaluation criteria revealed that B vitamins are essential for neurotransmitter synthesis and energy metabolism, making them critical for counteracting cognitive overload (27). Magnesium enhances these effects, reducing stress-induced cognitive impairments (20). The combination in FВ offers a practical delivery method, but optimal dosages and potential synergistic interactions require further investigation.

#### 3.5.3 Vision protection

Authors (19) state that lutein (10 mg) and zeaxanthin (2 mg) in FB filter blue light and reduce oxidative stress in the retina. A 12-month RCT involving 120 computer users demonstrated that daily intake of lutein and zeaxanthin increased macular pigment optical density by 20% and improved contrast sensitivity by 15%. Anthocyanins (50 mg) from blueberry extracts further alleviate visual fatigue by improving retinal microcirculation (28). For instance, daily consumption of 250 mL of FB containing lutein (10 mg), zeaxanthin (2 mg), and anthocyanins (50 mg) is expected to reduce visual fatigue symptoms (eye strain and dryness) in IT professionals by 30–40% over 3 months according to the Computer Vision Syndrome Questionnaire (CVS-Q). The effectiveness of visual fatigue symptoms was assessed based on the criteria presented in Table 12.

**Table 12:** Criteria for evaluating visual fatigue symptoms.

No.	Evaluation criteria	Effectiveness indicators
1	Objective indicators	Visual acuity (Snellen chart), contrast sensitivity (Pelli-Robson chart), and macular pigment optical density (heterochromatic flicker photometry).
2	Subjective indicators	CVS-Q for assessing symptoms such as eye strain, blurred vision, and headaches.
3	Study design	Double-blind RCTs with baseline and follow-up assessments to establish causality and account for variability in screen time.

It has been established that prolonged exposure to blue light from screens accelerates retinal damage, increasing the risk of age-related macular degeneration (29). Lutein and zeaxanthin protect photoreceptors, while anthocyanins enhance tear production, addressing oxidative stress and dry eye syndrome (28). FВ formulations should optimize bioavailability, potentially using nanoemulsions (23).

#### 3.5.4 Sleep improvement

The authors suggest that synbiotics combining *Bifidobacterium longum* 1714 and GOS enhance melatonin synthesis and reduce stress-related sleep disturbances. An RCT involving 80 adults with high digital workload showed that 8-week synbiotic intake reduced sleep latency by 20% and improved sleep quality by 25% according to the Pittsburgh Sleep Quality Index (PSQI) (30). For example, daily consumption of 200 mL of FB with synbiotics (*B. longum* 1714, 10^9^ CFU; GOS, 5 g) is expected to improve sleep quality by 20–30% (PSQI) in individuals with screen time >8 h/day over 2 months, enhancing daytime alertness and mood. The effectiveness of synbiotic use was assessed based on the criteria presented in Table 13.

**Table 13:** Criteria for evaluating synbiotic use.

No.	Evaluation criteria	Effectiveness indicators
1	Objective indicators	Polysomnography to assess sleep structure (e.g., REM and non-REM stages and sleep latency).
2	Subjective indicators	PSQI and sleep diaries to record perceived sleep quality and duration.
3	Study design	RCTs with actigraphy to monitor sleep patterns and control for lifestyle factors.

The analysis revealed that digital overload disrupts circadian rhythms, partly due to blue light exposure and stress-induced cortisol elevation (31). Synbiotics modulate the gut–brain axis, increasing the availability of melatonin and serotonin, which are critical for sleep regulation (32). Further studies are needed to investigate strain-specific effects and optimal synbiotic ratios.

#### 3.5.5 Prevention of hereditary stress

Adaptogens, such as ashwagandha (300 mg) in FB, reduce cortisol and anxiety. An RCT involving 60 parents showed that 12-week ashwagandha intake reduced anxiety by 30% [according to the Hospital Anxiety and Depression Scale (HADS)] and cortisol by 18% (18). It has been established that reducing parental stress can decrease the risk of mental disorders in children, given the relative risk of anxiety transmission of 3.09 (33). For example, daily consumption of 200 mL of FB containing ashwagandha (300 mg) and magnesium (200 mg) is expected to reduce parental anxiety by 25–35%, potentially lowering the risk of mental disorders in children by 10–15%, as assessed in longitudinal cohort studies. The effectiveness of adaptogen use was evaluated based on the criteria presented in Table 14.

**Table 14:** Criteria for evaluating adaptogen use.

No.	Evaluation criteria	Effectiveness indicators
1	Subjective indicators	Cortisol levels (in saliva or serum) and inflammatory markers (e.g., IL-6).
2	Subjective indicators	HADS and parental stress index to assess emotional wellbeing.
3	Study design	Hereditary cohort studies to track the mental health of parents and children over time.

The hereditary transmission of stress is a significant public health issue, as parental mental health impacts child development (7). The anxiolytic properties of ashwagandha, combined with the stress-modulating effects of magnesium, make FB a promising intervention (18). Long-term studies are needed to quantify its impact on children’s mental health.

#### 3.5.6 Synergistic effect

The hypothesis emphasizes the synergistic potential of FB components, which significantly enhances their protective effects against digital overload, achieving pronounced outcomes at minimal dosages. This synergism arises from the complementary actions of bioactive compounds that simultaneously target multiple physiological pathways associated with digital overload, such as stress responses, cognitive functions, the visual system, and sleep. This approach increases intervention efficacy and minimizes the risk of side effects, which is particularly important for long-term use in conditions of digital overload. A few examples of the synergistic potential of FB components are considered below.

*Magnesium and B vitamins:* Magnesium (200 mg) and B vitamins (thiamine 50 mg, pyridoxine 20 mg, and cobalamin 500 μg) synergistically enhance the synthesis of neurotransmitters such as serotonin and dopamine while supporting neuronal energy metabolism (34). An RCT involving 80 adults with cognitive overload showed that the combination of magnesium and B vitamins improved cognitive functions by 25%, compared to 15% with B vitamins alone (35). Magnesium further reduces cortisol levels, preventing stress-induced cognitive impairments, making this combination particularly effective for individuals with high digital workload (36).

*Lutein and omega-3 fatty acids*: Lutein (10 mg) and omega-3 fatty acids (500 mg) synergistically reduce inflammation and oxidative stress in the retina, protecting photoreceptors from blue light-induced damage. An RCT involving 120 computer users demonstrated that the combined administration of lutein and omega-3 reduced visual fatigue symptoms by 35% and improved contrast sensitivity by 20%, compared to 15 and 10% with each component alone (22). Omega-3 enhances lutein bioavailability, improving its accumulation in the macula, thereby underscoring the importance of their combined use (37).

*Probiotics, lutein, and omega-3*: A comprehensive RCT combining probiotics (*L. rhamnosus* GG, 10^9^ CFU), lutein (10 mg), and omega-3 (500 mg) showed a 35% reduction in visual fatigue, a 20% improvement in cognitive functions, and an 18% reduction in cortisol levels in 100 participants with screen time >8 h/day (22). Probiotics modulate the gut–brain axis, reducing systemic inflammation, which enhances the neuroprotective and anti-inflammatory effects of lutein and omega-3 (38). This combination demonstrated a synergistic effect, surpassing the results of single-component interventions.

*Ashwagandha and magnesium*: Ashwagandha (300 mg) and magnesium (200 mg) jointly regulate the hypothalamic–pituitary–adrenal axis, reducing cortisol levels and anxiety. An RCT with 60 parents showed that the combination of ashwagandha and magnesium reduced anxiety by 35%, compared to 25% with ashwagandha alone (39). Magnesium enhances the anxiolytic effect of ashwagandha by supporting neuronal stability and reducing stress-induced hyperactivity (40).

It has been established that daily consumption of FB (250 mL) containing a combination of lutein (10 mg), omega-3 (500 mg), probiotics (*L. rhamnosus* GG, 10^9^ CFU), magnesium (200 mg), B vitamins (thiamine 50 mg and pyridoxine 20 mg), and ashwagandha (300 mg) is expected to provide comprehensive mitigation of digital overload. The expected outcomes include the following: reduction of visual fatigue by 35–45% over 3 months; improvement in cognitive functions by 20–30% (per TMT and Stroop tests) over 12 weeks; reduction in anxiety by 30–40% over 8 weeks; improvement in sleep quality by 25–35% over 2 months; and reduction in cortisol levels by 15–20% and inflammatory markers (e.g., IL-6) by 10–15% (41).

These effects surpass the results of single-component interventions due to synergistic actions, allowing for lower dosages of individual components and minimizing the risk of side effects. Table 15 presents the evaluation of the synergistic effect of FB components.

**Table 15:** Evaluation of the synergistic effect of FB components.

No.	Evaluation criteria	Research indicators
1	Objective indicators	Biomarkers (cortisol, IL-6, and C-reactive protein), neuropsychological tests, ophthalmological parameters (contrast sensitivity and macular pigment density), and polysomnography (sleep structure).
2	Subjective indicators	Validated questionnaires (PSS-10, STAI, CVS-Q, PSQI, and HADS) to assess stress, visual fatigue, sleep, and anxiety.
3	Study design	Double-blind, placebo-controlled RCTs with multifactorial analysis to evaluate synergistic effects. Studies should include groups with single-component and combined interventions for efficacy comparison.

The synergistic effect of FB components is driven by their ability to simultaneously target multiple physiological pathways associated with digital overload. For instance, probiotics reduce systemic inflammation, enhancing the bioavailability and efficacy of lutein and omega-3, which, in turn, protect the neurons and photoreceptors (22, 42). Magnesium and B vitamins support neuronal function, amplifying the cognitive benefits of probiotics and adaptogens (33). Ashwagandha and magnesium jointly modulate stress responses, providing robust protection against technostress (18). These interactions enable significant effects at moderate dosages, making multi-component formulations economically and clinically effective. However, further studies are needed to determine optimal component ratios, bioavailability, and long-term safety. It is also critical to investigate the impact of synergistic combinations across diverse demographic groups, considering age, genetic factors, and levels of digital workload.

### 3.6 Practical implementation of the hypothesis

To translate the objective into practical interventions that confirm its protective potential against digital overload, the following strategies have been developed to integrate scientific evidence into real-world products and programs. These strategies aim to create effective, safe, and personalized solutions scalable for widespread use in conditions of digital overload. The practical implementation emphasizes the importance of synergistic formulations, advanced delivery technologies, individualized approaches, and rigorous scientific research to validate efficacy.

#### 3.6.1 Beverage formulation

The development of FB with carefully selected synergistic combinations of bioactive components is a key step in realizing the objective. Proposed formulations include lutein (10 mg), zeaxanthin (2 mg), omega-3 fatty acids (500 mg), probiotics (*Lactobacillus rhamnosus* GG, 10^9^ CFU), magnesium (200 mg), B vitamins (thiamine 50 mg, pyridoxine 20 mg, and cobalamin 500 μg), and the adaptogen ashwagandha (300 mg). These components were chosen based on their proven ability to mitigate the effects of digital overload, including stress, cognitive overload, visual fatigue, and sleep disturbances (19, 21). A daily serving of FB (200–250 mL) or FB (500 mL) provides an optimal dosage balanced for maximum efficacy and safety. An RCT with a combination of lutein, omega-3, and probiotics demonstrated a 35% reduction in visual fatigue and a 20% improvement in cognitive functions (22). Similarly, the combination of magnesium and B vitamins improved cognitive functions by 25% (34). These data confirm that multi-component formulations enhance protective effects, allowing for reduced dosages of individual components, which minimizes the risk of side effects. For example, the combination of ashwagandha and magnesium reduced anxiety by 35% compared to 25% for ashwagandha alone (18). These findings align with the conditions of the objective. Regular consumption of FB (250 mL/day) or FB (500 mL/day) with the specified components is expected to achieve the following: reduction in technostress by 25–35% over 8 weeks; improvement in cognitive functions by 20–30% over 12 weeks; reduction in visual fatigue by 35–45% over 3 months; and improvement in sleep by 25–35% over 2 months.

The efficacy of these formulations will be validated through RCTs incorporating biomarkers (cortisol and inflammatory markers), neuropsychological tests, ophthalmological parameters (such as contrast sensitivity), and questionnaires.

#### 3.6.2 Technological innovations

To enhance the bioavailability and efficacy of lipophilic components such as lutein, zeaxanthin, and omega-3, advanced delivery technologies, including nanoemulsions and liposomes, are proposed. Nanoemulsions increase the solubility and absorption of lipophilic compounds in the gastrointestinal tract, improving their delivery to target tissues (e.g., retina, brain) (23). Liposomes protect bioactive substances from degradation, ensuring stability and controlled release (23). These technologies allow for reduced dosages while maintaining high efficacy, which is critical for long-term use. Studies show that nanoemulsions of lutein increase its bioavailability by 60% compared to traditional forms, enhancing accumulation in the macula and protection against blue light (23). The liposomal delivery of omega-3 increases their plasma concentration by 40%, amplifying their anti-inflammatory effects (23). These technologies support the objective by ensuring the delivery of FB components to target systems, thereby enhancing their protective effects against digital overload. The use of nanoemulsions and liposomes in FB increases the bioavailability of lutein and omega-3 by 50–60%, allowing for a 20–30% reduction in dosages without loss of efficacy. This improves visual comfort by 40% and cognitive functions by 25% over a period of 3 months. The bioavailability of lipophilic components can be assessed through pharmacokinetic studies (measuring component concentrations in plasma and tissues), while efficacy can be evaluated through RCTs measuring biomarkers (cortisol and IL-6) and clinical outcomes (visual fatigue and cognitive functions).

#### 3.6.3 Personalization

To maximize the efficacy of FB, AI-based models are proposed to analyze individual data, including digital workload (screen time hours), age, gender, genetic predispositions (e.g., polymorphisms in genes related to vitamin metabolism), and lifestyle. AI models can create personalized formulations, optimizing dosages and component combinations. For example, individuals with high digital workload (>8 h/day) may require increased lutein and probiotic content, while older adults may need higher doses of vitamin D and calcium. Studies indicate that personalized dietary interventions based on genetic data improve clinical outcomes by 30% compared to universal approaches (43). The application of AI for FB personalization strengthens the objective by accounting for individual differences in susceptibility to digital overload. For instance, individuals with Methylenetetrahydrofolate reductase (MTHFR) polymorphisms may require higher B vitamin doses to support cognitive functions (44). Personalized FB developed using AI are expected to increase intervention efficacy by 25–40%, thereby achieving a 30–45% reduction in stress over 8 weeks, 25–35% improvement in cognitive functions over 12 weeks, and 40–50% reduction in visual fatigue over 3 months.

The efficacy of personalized formulations will be validated through RCTs using AI platforms to analyze data (genetics and digital workload). Comparisons with universal formulations will be conducted using biomarkers and questionnaires.

#### 3.6.4 Research priorities

To definitively validate the objective, placebo-controlled RCTs with robust endpoints are required. Studies should include the indicators specified in Table 16.

**Table 16:** Placebo-controlled RCTs with endpoints.

No.	Indicators	Measurement indicators
1	Biomarkers	Measurement of cortisol, inflammatory markers (IL-6 and C-reactive protein) to assess stress and inflammation.
2	Neuropsychological tests	TMT, Stroop, and Digit Span to evaluate cognitive functions.
3	Ophthalmological parameters	Contrast sensitivity and macular pigment density to assess visual function.
4	Questionnaires	PSS-10 (stress), CVS-Q (visual fatigue), PSQI (sleep), and HADS (anxiety) for subjective evaluation.
5	Study design	Double-blind RCTs lasting 8–12 weeks, including groups with single-component and combined interventions to assess synergism.

Preliminary RCTs confirm the efficacy of combined formulations (lutein, omega-3, and probiotics) in reducing visual fatigue by 35% and improving cognitive functions by 20% (22). Long-term studies will strengthen the hypothesis by providing data on safety and sustained effects. For example, a 12-month RCT with ashwagandha and magnesium demonstrated a sustained 35% reduction in anxiety (18). Additionally, RCTs will confirm that FB reduce stress biomarkers by 15–20%, improve cognitive functions by 20–30%, enhance visual comfort by 35–45%, and improve sleep by 25–35% compared to placebo. Long-term studies (6–12 months) will demonstrate the sustainability of effects and the absence of adverse reactions. To assess efficacy, a multifactorial analysis of RCT data will be conducted, including statistical significance (*p < 0.05*), as well as comparisons with single-component interventions to confirm synergism.

The practical implementation of the objective requires integrating scientific data into products accessible for widespread use. The multi-component nature of synergistic formulations supports the objective, providing comprehensive mitigation of digital overload (45). Nanoemulsion and liposome technologies enhance bioavailability, amplifying protective effects (46). Personalization through AI accounts for individual characteristics, maximizing efficacy (47). RCTs are critical for validating the hypothesis, providing objective data on clinical efficacy and safety. Future studies should also consider the economic feasibility and scalability of these solutions for diverse populations.

## 4 Conclusion

The objective that FB can mitigate the adverse effects of digital overload is convincingly supported by empirical evidence. Beverages enriched with probiotics, vitamins, minerals, adaptogens, and antioxidants effectively reduce stress, improve cognitive functions, protect vision, enhance sleep, and prevent the hereditary transmission of stress. The synergistic effects of multi-component formulations amplify their protective potential, offering a scalable solution to health challenges in the digital era. To ensure the efficacy of the proposed objective, focus should be placed on the following: optimizing dosages and component ratios through dose–response studies; conducting long-term RCTs to assess sustained benefits and safety; developing AI-based personalized nutrition platforms to enhance FB efficacy; and investigating new bioactive compounds (e.g., novel probiotic strains, adaptogens) to mitigate digital overload.

This evidence-based objective positions functional beverages as precise tools for mitigating digital stress through targeted nutrient delivery, systemic modulation of stress mechanisms, and physiological support tailored to age.

## Data Availability

The raw data supporting the conclusions of this article will be made available by the authors, without undue reservation.
